# Establishment of a Novel and Efficient *Agrobacterium*-Mediated *in Planta* Transformation System for Passion Fruit (*Passiflora edulis*)

**DOI:** 10.3390/plants10112459

**Published:** 2021-11-15

**Authors:** Hafiz Muhammad Rizwan, Qiang Yang, Ahmed Fathy Yousef, Xiaoxue Zhang, Yasir Sharif, Jia Kaijie, Meng Shi, Han Li, Nigarish Munir, Xuelian Yang, Xiaoxia Wei, Ralf Oelmüller, Chunzhen Cheng, Faxing Chen

**Affiliations:** 1 College of Horticulture, Fujian Agriculture and Forestry University, Fuzhou 350002, China; chrizwan51@gmail.com (H.M.R.); 1200305020@fafu.edu.cn (Q.Y.); Ahmedfathy201161@yahoo.com (A.F.Y.); 13647152752@139.com (X.Z.); 1180305003@fafu.edu.cn (J.K.); sm17720805309@163.com (M.S.); lh138158@126.com (H.L.); nigarish.munir@yahoo.com (N.M.); yangxuelian1995@163.com (X.Y.); ralf.oelmueller@uni-jena.de (R.O.); 2 Department of Horticulture, College of Agriculture, University of Al-Azhar (Branch Assiut), Assiut 71524, Egypt; 3 Institute of Oil Crops, College of Plant Protection, Fujian Agriculture and Forestry University, Fuzhou 350002, China; yasirsharif3336@gmail.com; 4 Fruit Research Institute, Fujian Academy of Agricultural Sciences, Fuzhou 350002, China; zhw7782352@sina.com; 5 Matthias Schleiden Institute, Plant Physiology, Friedrich-Schiller-University Jena, Dornburger Str. 159, 07743 Jena, Germany

**Keywords:** *Passiflora edulis*, dark incubation, parafilm wrapping, *in planta*, *Agrobacterium*-mediated transformation

## Abstract

Passion fruit (*Passiflora edulis*) is an important fruit crop with high economic value. Genetic engineering plays an important role in crop improvement with desired traits and gene functional studies. The lack of a simple, efficient, and stable transformation system for passion fruit has greatly limited gene functional studies. In this study, a simple and efficient *Agrobacterium*-mediated *in planta* transformation system for passion fruit was established, using *Agrobacterium* virulent strain EHA105 harboring the binary vectors pCAMBIA1301 and pCAMBIA1302 with GUS and GFP reporter genes. The system requires less time and labor costs than conventional transformation systems, and no additional phytohormones and sterile conditions are required. Regeneration efficiency of 86% and transformation efficiency of 29% were achieved, when the wounds were wrapped with Parafilm and the plants were kept in darkness for 15 days. Approximately 75% of the regenerated plants had a single shoot and 26% multiple shoots. The transformation was confirmed at the DNA and RNA levels as well as by GUS staining and GFP fluorescent measurements. The developed protocol will contribute to the genetic improvement of passion fruit breeding.

## 1. Introduction

Passion fruit (*Passiflora edulis*) originated from the Antilles of the Caribbean Sea and belongs to the *Passifloraceae* family. It has been widely cultivated throughout tropical and subtropical regions because of its attractive flavor and aroma, as well as nutrients including vitamins B1, B2, and C, and amino acids which are beneficial to human health [1]. The two main passion fruit cultivars, the yellow *Passiflora edulis* f. flavicarpa and the purple *P. edulis* Sims are grown and consumed globally due to their juice, flavor, and nutritional values [2]. Since the fruits contain high contents of phytochemicals, antioxidants, anti-inflammatory, and anti-cancer properties [3], the passion fruit industry and market are constantly growing worldwide because of rising consumer demands [4]. Genetic engineering made it possible to develop new crop varieties with desired traits [5], such as better pathogen resistance, higher productivity, nutritional value, and water use efficiency [6,7,8]. However, due to high ploidy, self-incompatibility, low genetic variability, and lack of disease resistant genes of the existing passion fruit varieties, the development of new and improved varieties is slow and costly [9]. The first transgenic passion fruit plants were generated in 1994 using *Agrobacterium-*mediated transformation [10]. Trevisan, Mendes [11] introduced the *passion fruit woodiness virus* (PWV) gene into the yellow cultivar (*P. edulis*), however, the transformation efficiencies were <0.5%. Comparable low transformation efficiencies were reported by Monteiro-Hara, Jadao [12] and Correa, Pinto [13] for the *cowpea aphid borne mosaic virus* (CABMV) coat protein gene. Additionally, Bunnag and Chamnanpon [14] reported low in vitro regeneration and transformation rates for purple passion fruit with *A. tumefaciens*. Tuhaise, Nakavuma [15] established an in vitro transformation protocol for Uganda’s yellow passion fruit and Asande, Omwoyo [9] generated transgenic hybrid passion fruit KPF4 with efficiencies below 1%. Only recently, da Silva, Pinto [16] established a sonication-assisted *A.-mediated* transformation protocol for anthers of wild passion fruit (*P. cincinnata* Mast.) with a transformation efficiency of 28%.

In general, *Agrobacterium mediated* and particle bombardment are used to introduce the target gene into a highly totipotent explant (callus, embryo, leaf or shoot apex), which are then followed by a tissue culture-based regeneration process [17,18]. *A*.-*mediated* transformation results in the incorporation of one or more copies of the full-length T-DNA into the host genome, resulting in stable gene expression, which could be affected by different factors including additional virulence genes (*Vir*) [19]. Although in vitro plant regeneration is suitable to a wide range of agricultural relevant plant species [20,21,22,23], it is time consuming and requires aseptic conditions [24]. Furthermore, the long regeneration phase can lead to morphological abnormalities and somaclonal variations [25,26]. In addition, many plant species are recalcitrant to in vitro regeneration [27,28,29]. In order to overcome these limitations and to achieve high transformation efficiencies, the non-tissue culture in vivo method of *Agrobacterium*-mediated transformation, called *in planta* transformation has become increasingly important.

*In planta* transformation with application of *Agrobacterium* inoculation on wounded plants often results in high transformation frequencies [30]. It has been used for many plant species, including *Arabidopsis thaliana* [31,32], wheat [33], rice [34], citrus [35], and alfalfa [36]. The method has been successfully developed for seeds, epicotyls, shoot apical nodes, flowers, and fruits as recipient tissues [37]. The non-sterile conditions are easier to handle, require less labor, time and costs, and result in fewer somatic cell clonal mutations [38]. The highly virulent *A. tumefaciens* strain EHA105 is most commonly used since it is more infectious than LBA4404 [39]. Marutani-Hert, Bowman [40], and Zhang, Zhang [35] showed that incubation of wounded seedlings in the dark is important for shoot regeneration after transformation in citrus. Furthermore, application of acetosyringone (AS) to the cultivation medium and a 3 days co-cultivation period enhance the transformation efficiencies [35,41]. However, to date, no *in planta* transformation system for passion fruit has been reported. We presented a transformation system, which includes seed germination, seedling decapitation, *Agrobacterium* inoculation of seedlings, wound wrapping, co-culture, selection, dark incubation, and transgenic line confirmation. The established *in planta* transformation protocol for passion fruit will allow to generate plants with improved genetic background, fruit quality, and plant resistance and provides a tool for gene functional studies.

## 2. Results

### 2.1. Influences of Dark Incubation and Parafilm Wrapping on Shoot Regeneration Efficiency

**Figure 1:**
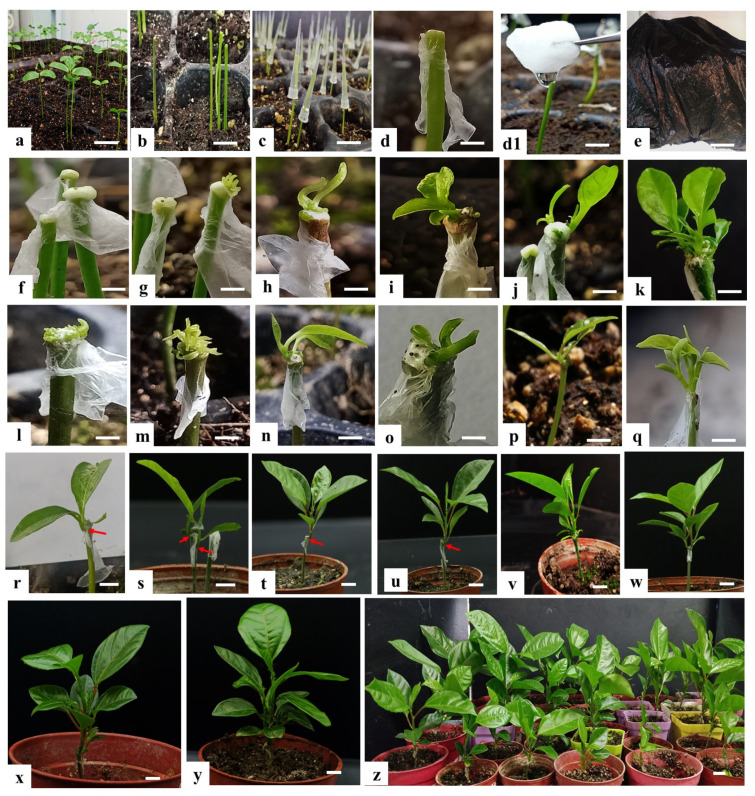
Procedure of Agrobacterium-medicated in planta transformation for passion fruit (*Passiflora edulis*). (**a**) Two–three weeks old passion fruit seedlings; (**b**) decapitated passion fruit seedlings; (**c**) *Agrobacterium* infection on the decapitated seedling wounded site; (**d**) wounds of *Agrobacterium* infected seedlings were wrapped with Parafilm; (**d1**) wounds of transformed seedlings were gently immersed in cotton balls saturated with 100 mg L^−1^ hygromycin for two to three times as selection marker/screening; (**e**) seedlings were kept under a black sheet for 15 days as dark incubation; (**f**) callus formed on wounded site; (**g**) buds sprouting from the newly formed callus; (**h**) single bud sprouted from the callus; (**i**) single bud elongated after 5 weeks of screening, sprouted from callus; (**j**) multiple buds sprouted from callus; (**k**) multiple buds elongated after 5 weeks of screening culture, sprouted from callus; (**l**,**m**) buds sprouting directly from xylem or regeneration occurs by direct organogenesis (without callus formation); (**n**) regeneration of single bud by direct organogenesis; (**o**) regeneration of multiple buds by direct organogenesis; (**p**) single bud elongated after 5 weeks of screening culture, regenerated by direct organogenesis; (**q**) multiple buds elongated after 5 weeks of screening culture, regenerated by direct organogenesis; (**r**) regenerated shoot from side of decapitated seedling; (**s**) regenerated shoots from side and below the decapitated seedling wounded spots; (**t**) regenerated shoot from side of decapitated seedling wound, after 3 months of screening culture; (**u**) regenerated shoot below the decapitated seedling wound, after 3 months of screening culture; (**v**) single shoot elongated after 3 months of screening culture; (**w**) multiple shoots elongated after 3 months of screening culture; (**x**) single shoot elongated after 5 months of screening culture; (**y**) multiple shoots elongated after 5 months of screening culture; (**z**) regenerated shoots from callus and without callus after 5 months of screening culture. (Scale bar = 1 cm).

The effects of wound wrapping with and without Parafilm on shoot regeneration after 0, 5, 10, 15, and 20 days in the dark incubation periods prior to culture under natural light conditions with additional 2 weeks from final time point of 20 days are shown in Table 1. The results of the present study indicated that within decapitated seedlings wrapped with Parafilm 66.53% regenerated shoots, while without Parafilm only 30.53% produced shoots. On the other hand, considering the effect of dark incubation on shoot regeneration of decapitated seedlings, 68.00% of the explants regenerated shoots, when the wounds were kept 20 days in the dark, whereas only 23.33% regenerated shoots without dark treatment (0 days). Without dark treatment, 37.3% of the explants regenerated shoots, when the wounds were wrapped with Parafilm, whereas only 9.3% regenerated shoots without the Parafilm. This suggests that keeping the wounds moist is an important parameter for shoot regeneration. Seedling wounds wrapped with Parafilm and kept for 15 days in dark incubation regenerated the maximum number of shoots 90% compared with the seedling wounds without Parafilm wrapping 41.3% under the same conditions. This clearly demonstrates the importance of the dark incubation for bud regeneration. Our results are consistent with those of Marutani-Hert, Bowman [42], and Zhang, Zhang [35] who showed that 2 weeks of dark incubation and Parafilm wrapping are essential for shoot regeneration. Based on the obtained results, we performed the transformation experiments using seedlings where the wounds were wrapped with Parafilm and incubated in the dark for 15 days (Figure 1).

### 2.2. Molecular Identification of Transgenic Plants

**Figure 2:**
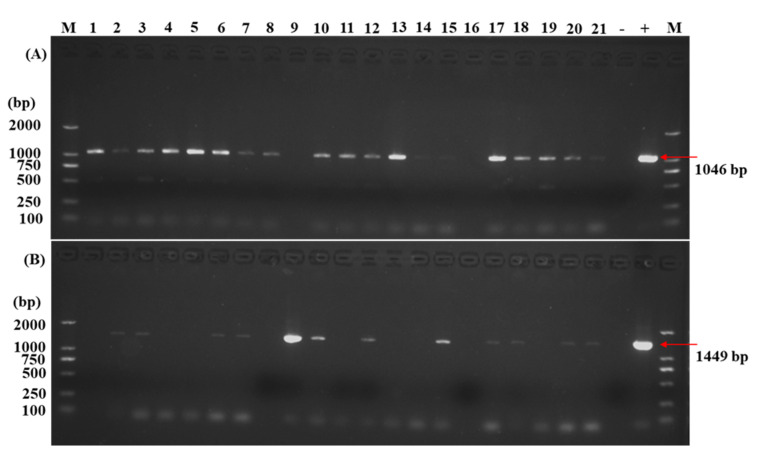
Detection of the transgenes in passion fruit. PCR analysis of genomic DNA of the transgenes using specific primers for (**A**) GUS gene and (**B**) GFP gene. M: DL2000 marker; 1-21: transgenic plants; -: control non-transgenic plants. +: pCAMBIA1301 and pCAMBIA1302 plasmid DNA as positive control.

The regeneration and *in planta* transformation efficiencies were studied after an additional 5–8 weeks from the final time point of the 15th day of dark incubation. Many adventitious shoots were discovered; most of them were regenerated from the newly formed callus (Figure 1f,g) but also regeneration occurs by direct organogenesis (without callus formation) (Figure 2l,m). The regeneration efficiency was 86%, with the majority of the regenerated plants having a single shoot, 74% (Figure 1h,i,n,p,v), and the rest had multiple shoots, 26% (Figure 1j,k,o,q,y. Table 2). In order to determine whether the transgene is integrated into the passion fruit genome, leaves from each shoot developed from adventitious buds were subjected to PCR analysis by using *GUS* (Figure 2A) and *GFP* (Figure 2B) gene-specific primer pairs. From 110 plants tested for the *GUS* gene and 105 plants for the *GFP* gene, 44 plants showed a *GUS*-specific PCR product and 18 plants a *GFP*-specific PCR product of the expected sizes. Based on these PCR analyses, an overall transformation efficiency of 29% was achieved (Table 2). In addition, the PCR amplification with specific primers for the *Agrobacterium Vir* genes confirmed the absence of the bacterium in the sampled tissues of the transgenic plants (Figure S1. Line 2–11).

### 2.3. GUS Staining Assays and Visualization of GFP Fluorescence in Transgenic Plants

**Figure 3:**
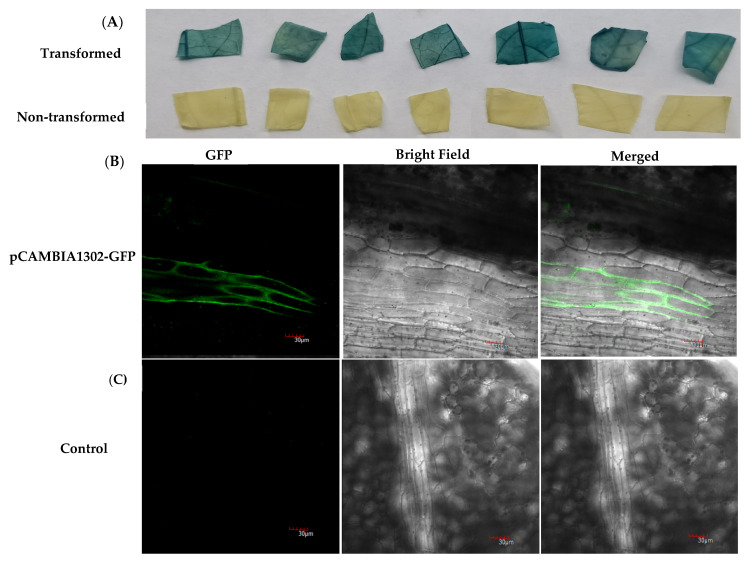
Histochemical GUS staining and visualization of GFP fluorescence in transgenic plants. (**A**) Histochemical β-glucuronidase (GUS) staining of leaf segments of different transgenic plants and non-transgenic control plants. (**B**,**C**) Visualization of GFP reporter gene in transgenic (pCAMBIA1302) and non-transgenic (control) passion fruit plant leaves under laser scanning confocal microscope. (**B**) GFP expression on transformed passion fruit plant leaf; (**C**) non-transformed plant leaf as control; all bars are 30 µm.

The leaves of the putative transgenic plants were also analyzed by GUS histochemical assay and GFP fluorescence (Figure 3). All *GUS*-positive tested plants and three non-transformed control plants were histochemically assessed for GUS staining. In total, 81.7% of the plants positively tested for the transformation with the *GUS* construct (Table 2) showed GUS staining while the control leaves were negative (Figure 3A), indicating that transgene was also expressed in the transformed passion fruits. Likewise, 56.7% of transformed plants with the *GFP* construct showed GFP fluorescence in the leaves, and the fluorescence was detectable in the entire cell compartment but not in the control plants (Figure 3B,C). We also found the variegated pattern of GUS histochemical staining assays in passion fruit transformed seedlings leaves and the variegated pattern of GUS histochemical staining assays shown in Figure S2. As shown in Figure S2, possible chimerism cannot be excluded a priori.

### 2.4. Expression Analysis of the GUS and GFP Genes by Quantitative Real-Time PCR

**Figure 4:**
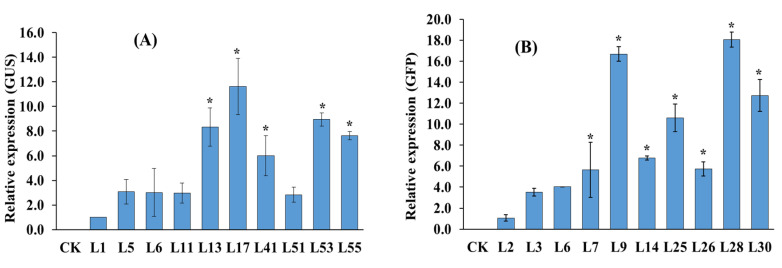
Relative expression assays of (**A**) GUS and (**B**) GFP genes by qRT-PCR in transgenic and non-transgenic (CK) plant leaves. Data are the mean ± standard deviation (SD) (*n* = 3), the measured values during the first transgenic line L1 and L2 were set to 1, *: indicates values which are significantly different from the first transgenic line value (ANOVA, *p* ≤  0.05), relative expression was calculated by 2^−ΔΔCT^ method.

The relative gene expression levels of *GUS* and *GFP* genes in 10 transgenic lines were tested by qRT-PCR. Transcripts can be detected in all transgenic lines, but not in the untransformed controls (Figure 4). However, the mRNA levels for the two reporter lines differed substantially in the leaves. For instance, the *GUS* transcript level in line ‘L17′ was 12 times higher (Figure 4A) than the level of ‘L1′ line and the *GFP* transcript level in the ‘L9′ and ‘L28′ was 17–18 times higher than the level of ‘L2′ line (Figure 4B), although their expressions were driven by the 35S promoter.

## 3. Discussion

The most desirable way to produce transgenic plants is to introduce target genes into the genomes of elite varieties with highly efficient plant regeneration systems [42]. Genetic engineering technology has been successfully applied to many plant species. For recalcitrant plant species such as passion fruit, the in vitro transformation and regeneration method is normally used [43]. Trevisan, Mendes [11], and Monteiro-Hara, Jadao [12] established in vitro regeneration and transformation with leaf disk explants from *Passiflora edulis* Sims. f. flavicarpa and achieved between 0.11% and 0.67% transformation efficiencies. Additionally, Correa, Pinto [13] achieved a low transformation efficiency of 0.89% using hypocotyl explants of *P. alata*. Similar results were obtained by Tuhaise, Nakavuma [15] and Asande, Omwoyo [9] between 0.456% and 0.67% with the KPF4 variety of *P. edulis* f. flavicarpa using leaf disk as explant for in vitro regeneration and transformation technique. Using an in vitro somatic embryogenesis sonication-assisted transformation system, da Silva, Pinto [16] achieved a transformation efficiency of 28.3% in *Passiflora cincinnata* anthers. In vitro transformation and regeneration systems are applicable for various plant species, but they are time-consuming and labor-intense, require aseptic cultivation conditions, and achieve low regeneration rates [16,20,21,22,23,24]. Furthermore, it is a high cost technology due to the requirement of sterile culture media, controlled sterile regeneration conditions, and long regeneration periods [44]. The simple and efficient *in planta Agrobacterium*-mediated transformation system for passion fruit described here requires non-sterile conditions, no addition of hormones, is less costly, highly efficient, and time saving. We showed that wrapping the wounds with Parafilm results in a substantial promotion of regenerated shoots presumably because the wounds were kept moist. Similar results were obtained by Zhang, Zhang [35] for *Citrus maxima.* The transformation and regeneration treatments usually comprise a dark incubation period which varies depending upon the genotypes. For instance, Clough and Bent [45] showed that covering *Arabidopsis* plants for 1 day after inoculation to maintain humidity increased transformation efficiency twofold. Priyadarshi and Sen [46] and Arzate-Fernandez, Nakazaki [47] reported that the dark conditions promote callus induction as well as the regeneration of somatic embryogenesis in *Lilium longiflorum* through auxins accumulation, which is degraded in light. We found that a dark period of 15 days produced the highest number of shoots in passion fruits, similar to observation by Zhang, Zhang [35] and Marutani-Hert, Bowman [40].

The plant transformation system requires a suitable marker gene for the recovery of transgenic plants [48]. We used hygromycin and achieved an effective and reliable transformation efficiency, similar to that reported by Asande et al. [9] for passion fruit and Zhang, Zhang [35] for *Citrus maxima.* Moreover, the bacterial noise for the selection of clean transgenic plants and to avoid concealed infections of *Agrobacterium* spp. were also assessed [49]. We confirmed our results by genomic PCR analyses, qPCR for transcript levels as well as GUS assays and GFP visualization. The results indicate that the intron-containing GUS and GFP genes were successfully expressed in the passion fruit.

In conclusion, the *in planta* transformation protocol described here is an alternative method for agricultural applications since it is more effective than previous in vitro tissue culture-dependent methods (Table 3), much easier to handle and cheaper. The *Agrobacterium*-mediated *in planta* transformation method in combination with the CRISPR-Cas9 technology will allow DNA manipulations at precisely chosen loci, as demonstrated by numerous examples in both model and crop plants [50].

## 4. Materials and Methods

### 4.1. Plant Material

The experiment was conducted in the Institute of Horticultural Plant Bioengineering, Fujian Agriculture and Forestry University, China. Healthy and freshly harvested mature seeds of passion fruit (*Passiflora edulis* Sims f. *edulis*) were provided by the Fruit Research Institute, Fujian Academy of Agricultural Sciences, Fuzhou, China. The seeds were moist incubated in muslin cloth for 2 weeks at 37 °C to allow germination and were then sown into seedling trays or plastic pots filled with peat moss and soil (2:1 ratio). Before sowing, the soil was thoroughly watered, and it was then watered once every 3 days after that. After 2–3 weeks of cotyledonary leaf emergence, the seedlings were used for *in planta* transformation (Figure 1a). All seedlings used in this study were kept in a greenhouse at 25 °C.

### 4.2. Agrobacterium Strain and Binary Vector

**Figure 5:**
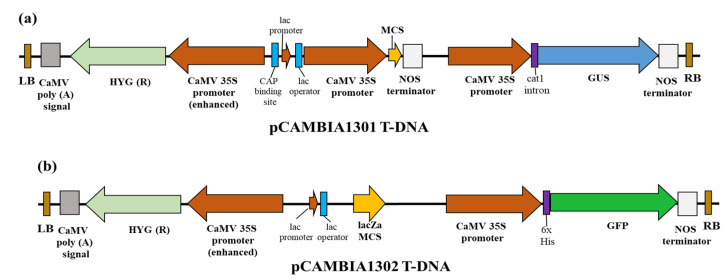
Expression vectors T-DNA regions. (**a**) pCAMBIA1301 plant expression vector T-DNA region containing GUS reporter and hygromycin resistance genes. (**b**) pCAMBIA1302 plant expression vector T-DNA region containing GFP reporter and hygromycin resistance genes.

The *Agrobacterium tumefaciens* strain EHA105 harboring the binary vector plasmids pCAMBIA1301 or pCAMBIA1302 was used to perform *in planta* transformation, which was kindly provided by Dr. Chun-zhen Cheng (Fujian Agriculture and Forestry University). The binary vector plasmid pCAMBIA1301 contained the β-glucuronidase (GUS) and pCAMBIA1302 the green fluorescent protein (GFP) reporter genes, respectively. Both plasmids contained the hygromycin B phosphotransferase gene (Hyg) as selection marker in their T-DNA regions. All genes were expressed under the control of the CaMV35S promoter and transcription was terminated by the nopaline synthase (NOS) terminator (Figure 5).

### 4.3. Agrobacterium Culture Preparation

*Agrobacterium* strain EHA105 harboring pCAMBIA1301 and pCAMBIA1302 stored at −80 °C was revitalized by streaking on Luria–Bertani (LB) agar medium (1% tryptone, 0.5% yeast extract, 1% sodium chloride (NaCl), 1.5% agar, and pH was adjusted to 7.2 with 0.1 N NaOH) Petri plates containing kanamycin (50 mg L^−1^) and rifampicin (50 mgL^−1^) antibiotics at 28 °C. A single bacterial colony obtained from streaked LB agar plates was grown overnight in 2 mL LB broth medium at 28 °C with orbital shaking of 150 rpm as starter cultures. The suspension was resuspended in 50 mL LB broth medium with the same antibiotics and grown by shaking at 250 rpm at 28 °C to obtain an optical density (OD_600_) of 1.0–1.3. The culture was centrifuged at 5000 rpm for 10 min, and the cell pellet was resuspended in 5% sucrose containing 0.01–0.05% (vol/vol) Silwet L-77 and acetosyringone (AS) at a concentration of 100 µM with an 0.6 OD_600_.

### 4.4. Experiment on Dark Incubation

The dark incubation experiment was carried out in the greenhouse before the final transformation, according to Zhang, Zhang [35] with slight modification. The primary leaves and apical meristem of passion fruit seedlings (2–3 weeks old) were removed with a scalpel, leaving the 1.5–2 cm long hypocotyl. In order to study the regeneration efficiency of dark culture, two kinds of wounded seedlings were investigated, one kind of injured seedling was wrapped with Parafilm, while other was not wrapped with Parafilm (without Parafilm) and then covered with a black plastic sheet for 0, 5, 10, 15, and 20 days at 25 °C, later shifted to natural light conditions for an additional 2 weeks (14 days) from the final 20th day dark incubation, 0 days means culture for 34 days in the light, 5, 10, 15, and 20 days mean culture for 5, 10, 15, and 20 days in the dark then materials were transferred to the light condition until the 34th day, the following parameters were assessed 2 weeks after the final 20th day dark incubation (total 34 days of culturing),regeneration efficiency (%) = No of shoots regenerated/total No of seedlings × 100; rate of single bud or shoot (%) = No of seedlings regenerated only one bud/total No of regenerated seedlings × 100; rate of multiple buds or shoots (%) = No of seedlings regenerated ≥ two buds/total No of regenerated seedlings × 100. At least 50 seedlings were used for each treatment and the experiment was performed three times.

### 4.5. In Planta Transformation of Passion Fruit Seedlings

The shoot apical meristem and primary leaves of passion fruit seedlings (2–3 weeks old) were removed with a scalpel (Figure 2b), and pipette tips (Figure 2c) were used for the *Agrobacterium* inoculation solution. The wounds were immersed with 40–60 µL inoculation solution pipetted with the pipette tip from the top onto the wounds, while seedlings immerged with 5% sucrose + 0.01–0.05% Silwet L-77 + 100 µM AS were used as control plants. After 40–50 min of submerging, the pipette tips were removed, and wounds were wrapped with Parafilm (Figure 2d) and covered with a black plastic sheet (Figure 2e). In order to obtain a higher transformation efficiency, 3 days after the *Agrobacterium* inoculation, the Parafilm was removed and the second inoculation (co-cultivation) was carried out by following the same steps as first time inoculation. After 3 days of co-culturing, Parafilm was removed and wounds of transformed seedlings were gently immersed in cotton balls saturated with 100 mg·L^−1^ hygromycin antibiotic for two to three times as selection marker (Figure 2(d1)). The selection marker controls the gene encoding the protein that allows transformed plants to survive in a toxic environment by providing resistance to the effects of antibiotics and helps monitor and select transformed plants. After immersion of wounds with 100 mg L^−1^ hygromycin, the wounds were wrapped again with Parafilm and placed under the black sheet for an additional 15 days (Figure 2e). After 15 days of dark incubation, the seedlings were then taken out from the sheet and moved to natural lighting in the greenhouse, to allow normal growth (Figure 2f–z). More than 25 seedlings were used for each transformation and the experiment was performed at least five times. A graphical representation of the whole passion fruit *in planta* transformation procedure is shown in Figure 2.

### 4.6. Identification of Putative Transgenic Plant Lines

#### 4.6.1. GUS Histochemical Staining Assay

After 5–8 weeks of transformation from the time point of the 15th day of dark incubation, the leaves (0.3–0.5 cm × 0.3–0.5 cm) of putatively transgenic passion fruit plants transformed with pCAMBIA1301 were collected for GUS histochemical assay according to Mahmood, Zeisler-Diehl [51] and leaves from non-transformed regenerated plants were used as controls. Leaf discs were immersed in GUS staining solution (100 mM sodium phosphate buffer (pH 7.2), 10 mM EDTA, 0.5 mM potassium ferricyanide, 0.5 mM potassium ferrocyanide, 2 mM X-Glu (5-bromo-4-chloro-3-indolyl-beta-D-glucuronic acid), 0.1% Triton X100, 20% methanol) and incubated overnight in the dark at 37 °C. The staining mixture was poured off, and subsequently, the plant material was washed repeatedly using 70% (*v*/*v*) ethanol for the elimination of chlorophyll contents, and blue stained leaf disks were photographed. Plants showing GUS blue spots were recorded as GUS positive plants. The transformation efficiency based on the GUS assays was calculated in (%) as No of GUS positive seedlings/total No of pCAMBIA1301 transgenic seedlings × 100.

#### 4.6.2. GFP Fluorescence Visualization

After 5–8 weeks of transformation from the time point of the 15th day of dark incubation, the leaves of putatively transgenic passion fruit plants transformed with pCAMBIA1302 were visualized for GFP expression. Leaves from regenerated shoots of non-transformed plants were used as controls. GFP expression was analyzed by using a laser scanning confocal microscopy (Olympus (Tokyo, Japan); FV1200) with excitation and emission wavelengths of 475–495 and 520–560 nm, respectively. The images were recorded in TIFF format. The transformation efficiency based on the GFP fluorescence assays was calculated in (%) as No of seedlings showing GFP fluorescence/total No of pCAMBIA1302 transgenic seedlings × 100.

#### 4.6.3. Isolation of Genomic DNA and PCR Analysis for Transgenic Lines

Genomic DNA for polymerase chain reaction (PCR) was isolated from 5–8 weeks (transformation from the time point of the 15th day of dark incubation) old regenerated transformants and non-transformed leaves using the cetyl trimethyl ammonium bromide (CTAB) method [52]. DNA quality and quantity were assayed on thermo scientific NanoDrop 2000 UV-Vis spectrophotometer (USA). The specific primers used in this study were designed by the Primer3Plus program (http://www.bioinformatics.nl/cgi-bin/primer3plus/primer3plus.cgi/, accessed on 11 April 2021). Primers used for GUS from pCAMBIA1301 were as follows: GUS-F: 5′-AATGGCGAATGCTAGAGCAG-3′; GUS-R: 5′-AAAGACTTCG CGCTGATACC-3′ (sequence 1046 bp). Primers used for GFP from pCAMBIA1302 were as follows: GFP-F: 5′-GCTGGCGTAATAGCGAAGAG-3′; GFP-R: 5′- GGGAAATTCGAGCTGGTC AC-3′ (sequence 1449 bp). In addition, the bacterial noise for the selection of clean transgenic passion fruit (*Passiflora edulis*) plants and to avoid concealed infections of *Agrobacterium* spp., we assessed the background bacterial noise using the *Vir*D1, *Vir*D4, *Vir*E2, and *Vir*A/G gene specific primers by PCR. The sequences of these genes were downloaded from the National Center for Biotechnology Information (NCBI database). Primers used for *Vir*D1-F: 5′-ACCTCGTGTGACATTGCCAT-3′; *Vir*D1-R: 5′-CAAGGCGTCTTTCAGCATCG-3′ (420 bp), *Vir*D4-F: 5′-ACTGCCTGGTAAAGACGAGC-3′; *Vir*D4-R: 5′-TGTTGAATCCATGTCGC CCA-3′ (610 bp), *Vir*E2-F: 5′-CAAATCTAGCGACCCTGCCA-3′; *Vir*E2-R: 5′-GAGGATATCGGGGAGCAACG-3′ (565 bp); *Vir*A/G-F: 5′-AGGAAACCCGAAAGTCGACC-3′ and *Vir*A/G-R: 5′-CAGTTATCATATGCCGCGCG-3′ (618 bp). PCR amplification was carried out in 25 µL reaction mixture containing 100 ng genomic DNA, 1.25 µL of each primer (100 µM), 12.5 µL 2x Taq PCR MasterMix (Tiangen, China), and 9 µL sterilized MilliQ water. PCR was carried out by using T100TM Thermal cycler (Bio-Rad, Singapore) with the following conditions: 94 °C for 5 min pre-denaturation, followed by 34 cycles for 30 s at 94 °C, 30 s annealing at 60 °C, 1 min/kb extension at 72 °C, final extension for 5 min at 72 °C, and held at 12 °C. The PCR products (6 µL) were analyzed by running the sample on a 1.5% agarose gel in 1x TAE (Tris-acetate-EDTA) buffer, visualized under a UV transilluminator (Peiqing, model: JS-680D, China), and photographed by the gel documentation system. Statistics of transgenic plants based on electrophoresis detection conversion rate were calculated as follows: Transformation efficiency (%) = No of PCR positive seedlings/total No of seedlings × 100.

### 4.7. RNA Extraction for qRT-PCR Analysis

RNA was extracted from PCR-positive plant leaves using the Tiangen mini RNA extraction kit (Tiangen, China), and quantitative real-time PCR (qRT-PCR) was performed to analyze the expression of *GUS* and *GFP* genes. A Thermo Scientific NanoDrop 2000 UV-Vis spectrophotometer (USA) was used to determine the quantity and quality of total RNA (A_260/230_ and A_260/280_). Complementary DNA (cDNA) was synthesized using 1 µg of total RNA by Takara PrimeScript ^TM^ RT Reagent Kit with gDNA eraser (TAKARA, China). Specific primers used for qRT-PCR: qPCR-GUS-F: 5′-AACCGTTATTACGGATGGTATGT C-3′; qPCR-GUS-R: 5′-GCA CACTGATACTCTTCACTCCAC-3′; qPCR-GFP-F: 5′-AAGA ACTTTTCACTGGAGTTGTC C-3′; qPCR-GFP-R: 5′-CGGAACAGGTAGTTTTCCAGTA GT-3′; and 60S-F: 5′-AGGTGGGTAACAGGATTATC-3′; 60S-R: 5′-TGGCTGTCTTTTGG TGCTG-3′ as housekeeping gene [53]. The qRT-PCR reaction was performed on LightCycler^®^ 96 (Roche Applied Science, Penzberg, Germany) in a 20 µL reaction mixture composed of 10 µL TB Green premixed enzyme solution (TAKARA), 1.0 µL each of the forward and reverse primers (100 μM), 1 µL cDNA, and 7 µL ddH_2_O. The qRT-PCR conditions included preincubation at 95 °C for 30 s, followed by 45 cycles at 95 °C for 10 s, and 60 °C for 30 s. Each reaction was repeated three times. The relative gene expression levels were calculated using the 2^−ΔΔCT^ method [54], and the measured value of the first transgenic line of L1 and L2 (Figure 5) was set to relative expression level of 1.0.

### 4.8. Data Analysis

The data were analyzed with SPSS software version 12.0, and statistical significance was determined using analysis of variance (ANOVA). Least significant difference (LSD; *p* < 0.05) test was used to determine the significance of differences among means, all the percentage data was transformed to arcsine values prior to the statistical analysis.

## 5. Conclusions

In this study, a simple and efficient *Agrobacterium*-mediated *in planta* transformation system was established in commercial passion fruit for the first time. This system includes seedling preparation, decapitation, *Agrobacterium* inoculation of seedlings, Parafilm wrapping, 15 days of the dark incubation period promoting high shoot regeneration, and transformation efficiencies. The successful regenerated transgenic passion fruit plants were confirmed by PCR analysis at the DNA and RNA levels, as well as by GUS assays and GFP fluorescent measurements. The developed protocol will be useful for the genetic improvement of passion fruit against stresses and fruit quality as well as gene functional studies.

## Figures and Tables

**Table 1:** Effect of dark incubation periods, Parafilm wrapping, and the interaction on shoot regeneration. Decapitated seedlings wrapped with or without Parafilm were kept in the dark for 0, 5, 10, 15, and 20 days prior to culture under natural light conditions for another 2 weeks.

Treatments		With Parafilm	Without Parafilm	Mean Factor B (Dark Incubation)
**Dark incubation periods**	**0 day**	37.33 ± 3.89 ^d^	9.33 ± 4.42 ^e^	23.33 ± 15.39 ^d^
	**5 days**	47.33 ± 9.04 ^c^	17.33 ± 4.90 ^e^	32.33 ± 17.57 ^c^
	**10 days**	70.67 ± 6.46 ^b^	36.00 ± 4.42 ^d^	53.33 ± 19.18 ^b^
	**15 days**	90.00 ± 5.96 ^a^	41.33 ± 5.42 c ^d^	65.67 ± 26.34 ^a^
	**20 days**	87.33 ± 6.46 ^a^	48.67 ± 6.86 ^c^	68.00 ± 21.55 ^a^
	**Mean Factor A** **(Parafilm)**	66.53 ± 22.53 ^a^	30.53 ± 16.06 ^b^	
LSD Factor A (*p ≤* 0.05)		7.025		
LSD Factor B (*p ≤* 0.05)		5.832		
LSD Interaction A × B (*p ≤* 0.05)		8.248		

Note: data are represented as mean ± SD (standard deviation) of regenerated/total seedling. The different letters in this table mean significant at *p ≤* 0.05 level using LSD Test.

**Table 2:** Rate of regeneration, transformation, single or multiple buds of *Passiflora edulis* after *in planta* transformation and regeneration. Data are represented as mean ± SD (standard deviation) in%.

	No. of Plants	%
Regeneration efficiency ^a^	215/250	86.0 ± 4.0
Overall transformation efficiency ^b^	62/215	28.8 ± 4.6
GUS assays efficiency ^c^	36/44	81.7 ± 5.4
GFP fluorescence efficiency ^d^	10/18	56.7 ± 8.2
Single bud rate ^e^	47/62	74.4 ± 8.8
Multiple bud rate ^f^	16/62	25.7 ± 4.5
PCR GUS-positive efficiency ^g^	44/110	40.0 ± 5.0
PCR GFP-positive efficiency ^h^	18/105	17.1 ± 5.9

^a^ Regeneration efficiency (%) = No of seedlings regenerated shoots/total No of seedlings × 100. ^b^ Overall transformation efficiency (%) = No of PCR positive seedlings/total No of regenerated seedlings × 100. ^c^ GUS assays efficiency (%) = No of seedlings showing GUS blue/total No of pCAMBIA1301 transgenic seedlings × 100. ^d^ GFP fluorescence efficiency (%) = No of seedlings showing GFP fluorescence/total No of pCAMBIA1302 transgenic seedlings × 100. ^e^ Single bud rate (%) = No of seedlings regenerated only one bud/total No of regenerated seedlings × 100. ^f^ Multiple buds rate (%) = No of seedlings regenerated ≥ two buds/total No of regenerated seedlings × 100. ^g^ PCR GUS-positive efficiency = No of seedlings showing GUS specific bands/total No of pCAMBIA1301 regenerated seedlings × 100. ^h^ PCR GFP-positive efficiency = No of seedlings showing GFP specific bands/total No of pCAMBIA1301 regenerated seedlings × 100.

**Table 3:** Previous reports about the genetic transformation in *Passiflora* from different explant sources by in vitro methods and efficiencies.

Cultivars	Vector and *Agrobacterium* Strain	EXPLANTS	Methods	Efficiency (%)	References
***Passiflora edulis* Sims. f. flavicarpa. IAC-275 and IAC-277.**	pCAMBIA2300; EHA105	Leaf disks	In vitro regeneration and transformation	0.11–0.21	[11]
***Passiflora edulis* Sims. f. flavicarpa, IAC-275 and IAC-277.**	pCAMBIA2300; EHA105	Leaf disks	In vitro regeneration and transformation	0.19–0.67	[12]
***Passiflora alata***	pCABMVdsCP; EHA105	Hypocotyls	In vitro regeneration and transformation	0.89	[13]
***Passiflora edulis* f. flavicarpa.**	pCAMBIA2301; JM109	Leaf disks	In vitro regeneration and transformation	0.456	[15]
***Passiflora edulis* f. edulis × *Passiflora edulis* f. flavicarpa = KPF4.**	pCAMBIA1301; LBA4404	Leaf disks	In vitro regeneration and transformation	0.67	[9]
***Passiflora cincinnata* Mast.**	pCAMBIA1304; LB4404	Anthers	In vitro somatic embryogenesis and sonication-assisted transformation	28.26	[16]

## Data Availability

Not applicable.

## Supplementary Materials

The following are available online at https://www.mdpi.com/article/10.3390/plants10112459/s1, Figure S1: VirD1, VirD4, VirE2, and VirA/G gene specific primers were used to evaluate the background bacterial noise in passion fruit (Passiflora edulis) transgenic plants by PCR amplification. Figure S2. Pattern of GUS histochemical staining assays in passion fruit transformed (a-g) and non-transformed seedlings (leaf parts). Table S1. Analysis of Variance Analysis (ANOVA) table for regeneration.
