# Supplementary material for: Establishment of a Novel and Efficient Agrobacterium-Mediated in Planta Transformation System for Passion Fruit (Passiflora edulis)
Source: Plants (Basel). 2021 Nov 15;10(11):2459. doi: 10.3390/plants10112459 (PMC8621743; doi:10.3390/plants10112459)
Supplement: Supplementary file 1 [file plants-10-02459-s001.zip › plants-1415011-supplementary.pdf]

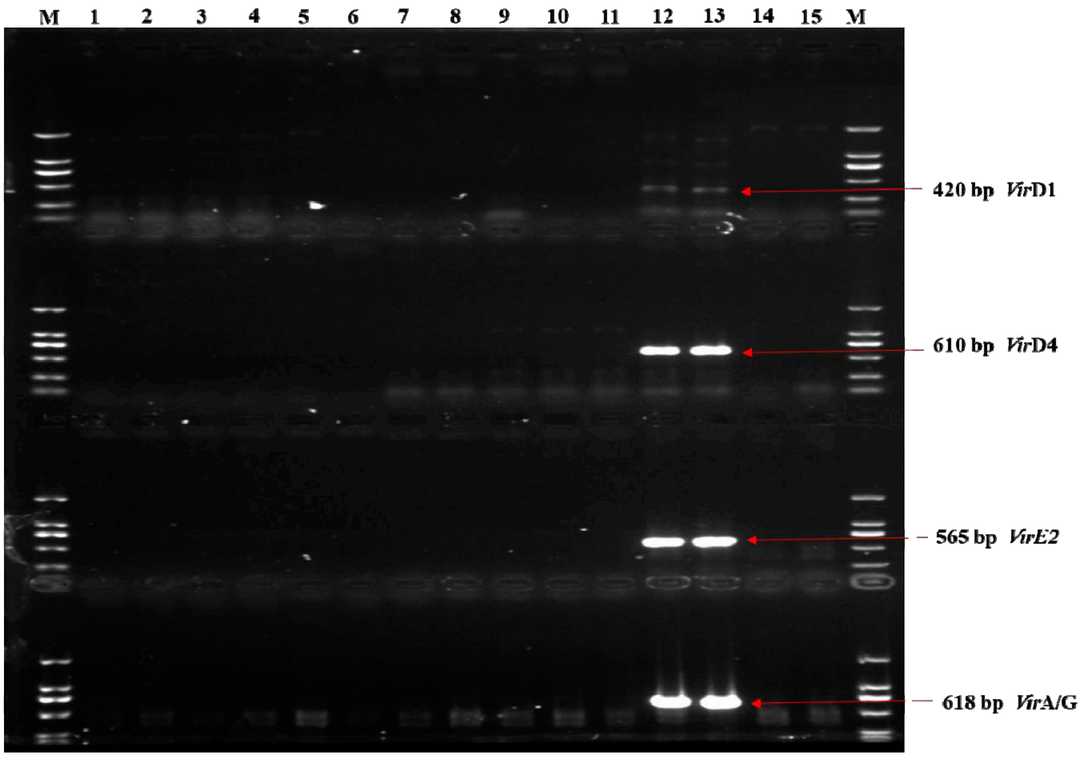

Figure S1. *Vir*D1, *Vir*D4, *Vir*E2, and *Vir*A/G gene specific primers were used to evaluate the background bacterial noise in passion fruit (*Passiflora edulis*) transgenic plants by PCR amplification. M= 2kb marker; Line 1 contains the negative control (template DNA from non-transformed passion fruit plants); Line 2-6 contains pCAMBIA1301 transgenic plants DNA; Line 7-11 contains pCAMBIA1302 transgenic plants DNA; Line 12 contains positive control from pCAMBIA1301 DNA; Line 13 contains positive control from pCAMBIA1302 DNA; Line 14-15 contains the water control (no template DNA).

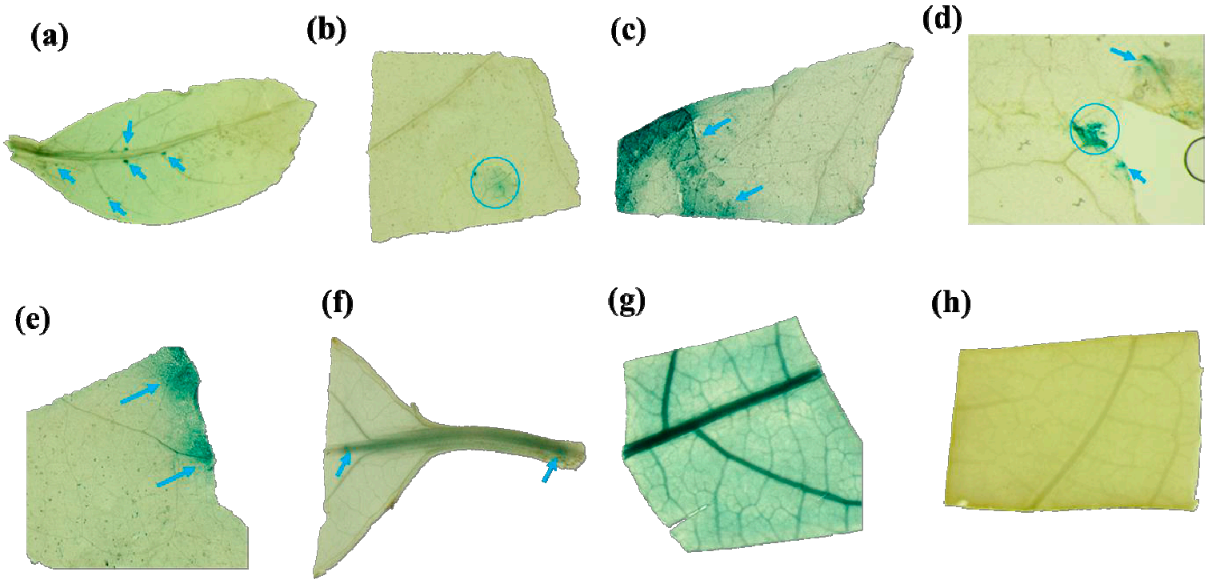

Figure S2. Pattern of GUS histochemical staining assays in passion fruit transformed (a-g) and non-transformed seedlings (leaf parts). a-f) variegated GUS staining patterns were detected in different leaf parts of the passion fruit transformed seedling, g) a unique GUS staining pattern detected in passion fruit transformed seedling leave, h) no GUS staining was detected in non-transformed passion fruit seedlings leaves. Different GUS patterns are highlighted by arrows and circles.

Table S1. Analysis of Variance Analysis (ANOVA) table for regeneration.

**Source**	**DF**	**SS**	**MS**	**F**	**P**
Replication	4	145.9	36.5		
Parafilm	1	16199.3	16199.3	202.44	0.0001
Error Replication*Parafilm	4	320.1	80.0		
Days	4	15931.0	3982.8	97.15	0.0000
Parafilm*Days	4	673.3	168.3	4.11	0.0085
Error Replication*Parafilm*Days	32	1311.9	41.0		
Total	49	34581.4			
